# Addition of an online, validated family history questionnaire to the Dutch FIT-based screening programme did not improve its diagnostic yield

**DOI:** 10.1038/s41416-020-0832-8

**Published:** 2020-04-20

**Authors:** Victorine H. Roos, Frank G. J. Kallenberg, Manon van der Vlugt, Evelien J. C. Bongers, Cora M. Aalfs, Patrick M. M. Bossuyt, Evelien Dekker

**Affiliations:** 1 Department of Gastroenterology and Hepatology, Cancer Centre Amsterdam, Amsterdam Gastroenterology and Metabolism, Amsterdam UMC, University of Amsterdam, Meibergdreef 9, Amsterdam, Netherlands; 2 Foundation of Population Screening Mid-West, Amsterdam, Netherlands; 3 Department of Clinical Genetics, Amsterdam UMC, University of Amsterdam, Meibergdreef 9, Amsterdam, Netherlands; 4 Department of Clinical Epidemiology, Biostatistics and Bioinformatics, Amsterdam UMC, University of Amsterdam, Meibergdreef 9, Amsterdam, Netherlands

**Keywords:** Population screening, Colorectal cancer, Genetic testing, Cancer screening, Population screening

## Abstract

**Background:**

Faecal immunochemical testing (FIT) is suboptimal in detecting advanced neoplasia (AN). To increase the sensitivity and yield of a FIT-based screening programme, FIT could be combined with risk factors for AN. We evaluated the incremental yield of adding a family history questionnaire (FHQ) on colorectal cancer (CRC) and Lynch syndrome-associated tumours to the Dutch FIT-based screening programme.

**Methods:**

Six thousand screen-naive individuals, aged 59–75 years, were invited to complete a FIT (FOB-Gold, cut-off 47 µg Hb/g faeces) and a validated online FHQ. Participants with a positive FIT and/or positive FHQ, confirmed after genetic counselling, were referred for colonoscopy. Yield of detecting AN per 1000 invitees for the combined strategy was compared with the FIT-only strategy.

**Results:**

Of the 5979 invitees, 1952 (32.6%) completed the FIT only, 2379 (39.8%) completed both the FIT and FHQ and 95 (1.6%) completed the FHQ only. Addition of the FHQ to FIT-based screening resulted in one extra case of AN detected after 16 additional colonoscopies, resulting in a yield of 19.6 (95% CI, 16.4–23.5) for the combined strategy versus 19.5 (95% CI, 16.3–23.3) for the FIT-only strategy (*p* = 1.0).

**Conclusions:**

The addition of an FHQ to one round of FIT screening did not increase the detection of AN compared with FIT only (ClinicalTrials.gov NCT02698462).

## Background

Colorectal cancer (CRC) is the third most prevalent cancer in the Netherlands.^1^ CRC predominantly originates from adenomas.^2^ As the progression of adenomas to cancer takes 10–15 years, there is a long window of opportunity for intervention.^2^ Colonoscopy enables detection and treatment of adenomas and early cancer, reducing both CRC-related incidence and mortality.^3^

Faecal immunochemical testing (FIT) is a screening method that selects individuals at high risk of having advanced adenomas or CRC, the combination referred to as advanced neoplasia (AN). Screening participants with a positive FIT are subsequently invited for colonoscopy. FIT has a low burden, facilitating participation in FIT-based screening. In the Netherlands, the participation rate is around 72%.^4^ Yet, since the sensitivity of FIT is not perfect, not all individuals with AN are detected in one screening round. Depending on the faecal haemoglobin concentration cut-off level and brand, pooled sensitivity was 79% for CRC and 6–56% for AN.^5,6^

To increase the detection rate of AN in FIT-based screening, FIT could be combined with risk factors for AN. Studies have reported that a false-negative FIT result was more often observed in participants with a family history of CRC than in those without.^7,8^ In this context, a positive family history is defined by the diagnosis of a hereditary CRC syndrome, such as Lynch syndrome, and/or the so-called “familial CRC syndrome”, defined by the number and age of relatives with CRC but which does not yet have a known genetic basis. Of all the CRC cases, 15–30% seem to have such a familial risk and 2–5% are related to a hereditary CRC syndrome.^9^

International guidelines recommend individuals with a positive CRC family history to undergo regular colonoscopy surveillance instead of participating in FIT-based screening programmes.^10,11^ Colonoscopy surveillance has shown to reduce CRC-related mortality for individuals with Lynch syndrome by 72% and up to 81% for those with a familial CRC syndrome.^12,13^ However, colonoscopy uptake in individuals with a positive family history for CRC varies widely (12–51%).^14^ Furthermore, only 12–33% of CRC patients and their relatives who qualify for referral to a clinical geneticist are actually referred.^15,16^ Contrary to the guideline recommendations, many individuals with a hereditary or familial CRC syndrome do not receive periodic colonoscopy surveillance. Instead, they will be invited for screening whenever an organised population-based screening programme is in place.

In the Australian FIT-based population screening programme, 4% of the participants had a positive family history of CRC or polyps, warranting colonoscopy surveillance.^17^ Only 50% of those individuals had recently undergone surveillance.^17^ Another study showed that offering colonoscopy surveillance to participants with a positive FIT or CRC family history increased sensitivity in detecting AN, at the expense of specificity.^18^ In this study, the definition of a positive CRC family history was limited to first-degree relatives with CRC, regardless of their age.^18^

The limited sensitivity of FIT and the limited identification of individuals with a positive family history call for the exploration and evaluation of simple online questionnaire to improve the current situation. We designed a study to evaluate the incremental yield of adding a validated online family history questionnaire (FHQ) on CRC and Lynch syndrome-associated tumours to the Dutch organised FIT-based screening programme.^19^ In this study, participants with a positive FIT and/or a positive FHQ were offered a colonoscopy. We compared the yield of this combined strategy with that of the FIT-only screening strategy. As a secondary aim, we studied the identification of individuals with a hereditary or familial CRC syndrome by the FHQ and the subsequent referral rates for genetic counselling and colonoscopy.

## Methods

### Study population and study design

We performed a prospective population-based CRC screening trial embedded in the national CRC screening programme. Six thousand screening-naive individuals, aged from 58–59 to 74–75 years, were invited to sample an FIT and to complete an FHQ. Invitees were selected from four areas in the province of North Holland based on their year of birth (1941, 1945, 1953, 1955 and 1957). These areas reflected the nationwide CRC screening programme in 2014 regarding socioeconomic status (SES), ethnicity and participation rates. Within these areas, invitees were selected with an age and sex distribution comparable to that of the national CRC screening programme population in 2016. SES was based on postal code areas. There were no specific exclusion criteria for participation in the study.

### Study procedures

#### Invitation for FIT and FHQ

In line with standard procedure of the national FIT-based screening programme, all invitees received an FIT pre-announcement letter. This letter contained a study invitation letter including a personal login code and information leaflet. Using this personal login code, a validated online FHQ could be filled out after informed consent was given.

The Dutch CRC screening programme provided all invitees with an FIT kit by postal mail 14–21 days after the pre-announcement. Invitees who declined participation in FIT screening were asked to inform the screening organisation. Seven-to-14 days after sending the FIT kit, a reminder study invitation letter was sent. Invitees who had not responded to the FIT invitation received a reminder 56 days after the pre-announcement.

FIT analysis and communication with participants regarding the FIT result were according to the logistics of the national CRC screening programme. The positivity threshold of the FIT was set at 47 µg haemoglobin/g faeces in the Dutch national CRC screening programme. All participants received a letter with the FIT result, either a positive or negative result, within 7–14 days after returning the device. Participants with a positive test result received an appointment for an intake visit for colonoscopy.

#### Family history questionnaire

The online FHQ was based on a previously validated questionnaire, with high sensitivity and specificity in identifying individuals qualifying for referral because of suspected Lynch syndrome or familial CRC syndrome (Supplementary Information: Family History Questionnaire in Dutch).^19^ Questionnaire responses were automatically evaluated against the national referral criteria for genetic testing for Lynch syndrome and surveillance colonoscopies in case of a familial CRC syndrome (Supplementary Information Table 1). As an additional quality control measure, all generated results were manually checked by the research fellow (V.H.R.). A second research fellow (F.G.J.K.) performed additional verification of 10% of the sample.

In case an FHQ participant returned the FIT, FHQ results were sent to the participant within 7 days after receiving the FIT result. For participants who did not return the FIT, the FHQ result was sent 15 weeks after the pre-announcement letter. All participants who received a positive FHQ result were contacted by telephone by the research fellow (V.H.R.) within 10 days after receiving the FHQ result. To identify potential false-positive results, this trained researcher verified all answers with the participant. Once the positive CRC family history was confirmed, an intake at the Department of Clinical Genetics was arranged. During this intake, a clinical geneticist or genetic counsellor decided whether genetic testing or a surveillance advice was indicated.

#### Colonoscopy

All participants with a positive FIT and/or a positive FHQ, confirmed after genetic counselling, were referred for colonoscopy. Colonoscopies after a positive FIT and/or a confirmed familial CRC syndrome were performed in one nationally accredited colonoscopy centre (Bergman Clinics Amsterdam), unless the participant preferred another centre. In participants with Lynch syndrome, a colonoscopy was performed in a dedicated tertiary colonoscopy centre (Academic Medical Centre). All colonoscopies in participants with a positive FIT were performed by accredited screening gastroenterologists, according to the national CRC screening programme standards, who had performed at least 200 colonoscopies per year.^20^ Colonoscopies in those with a familial or hereditary CRC syndrome were performed by gastroenterologists with similar adenoma detection rates. Colonoscopies were conducted according to international standard quality criteria.^21^ Resected lesions were evaluated by experienced pathologists according to the World Health Organisation classification and Vienna criteria.^22,23^ Advanced adenomas were defined as adenomas having a diameter ≥10 mm and/or with ≥25% villous component and/or high-grade dysplasia. Depending on the findings, surveillance colonoscopies were advised according to national guideline criteria.^24^

### Statistical analysis

The primary study outcome was diagnostic yield, defined as the number of participants in whom AN was detected relative to the number of invitees. We compared the diagnostic yield of a combined approach, selecting participants for colonoscopy based on FIT and family history, to that of (hypothetical) selection based on FIT only, in the same group of participants. Secondary outcomes were participation rate and positive predictive values. The positive predictive value of the screening instrument was defined as the number of participants in whom AN was detected relative to the number of participants testing positive. Differences in diagnostic yield and participation rate were tested for statistical significance using McNemar test statistic. A difference in positive predictive value between the two strategies was assessed for statistical significance with Chi-square test statistic.

Additional outcomes were positivity rate, number of colonoscopies performed, and detection rate. A significance level of 5% was used in all statistical tests. All statistical analyses were performed using R Studio version 1.1.383.

### Sample size calculation

Our sample size calculation was based on the anticipated gain in diagnostic yield when using the combined strategy.^18^ With a study group of 6000 individuals, we expected to detect 130 individuals with AN after a positive FIT (21.7 per 1000 invitees). By adding the FHQ, we anticipated to detect an additional number of 11 individuals with AN (23.5 per 1000 invitees): an increase in diagnostic yield of 1.8 per 1000 invitees. We then would have a power of at least 91% in rejecting the null hypothesis of no gain, using McNemar test statistic.

### Ethical considerations

Ethical approval for this study embedded in the national screening programme was obtained from the Dutch National Health Council. The study was performed in accordance with the Declaration of Helsinki.^25^

## Results

### Screening population

**Fig. 1:**
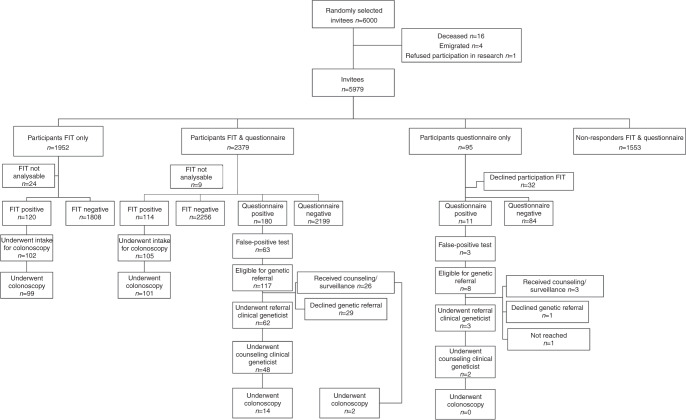
Flowchart of study population.

Of the 6000 initially selected, randomly sampled invitees, 16 were deceased, 4 had emigrated prior to the invitation for the study and 1 individual refused participation in research. Between April 2016 and January 2017, 5979 invitees received both an FIT and the FHQ (Fig. 1).

### Participation

Of the 5979 invitees, 1952 (32.6%) completed the FIT only, 2379 (39.8%) completed both the FIT and the FHQ and 95 (1.6%) invitees completed the FHQ only; 1553 (26.0%) invitees neither returned an FIT nor completed the FHQ.

Table 1 lists key characteristics of invitees and four subgroups: participants who completed FIT, FHQ or both and non-participants. There were more people born in 1941 in the FIT-only group, compared to the other participants. The FIT-only group had more females, whereas there was a small majority of males among non-participants. In the small group who completed the FHQ only, without returning the FIT, more participants could be classified in the very high SES category, compared to the other groups.

**Table 1:** Demographics.

	Invitees, *n* = 5979	Participants FIT only, *n* = 1952	Participants FIT and FHQ, *n* = 2379	Participants FHQ only, *n* = 95	Non-participants, *n* = 1553	*p* Value^a^
Year of birth						
1941	769 (12.9%)	290 (14.8%)	256 (10.8%)	10 (10.5%)	213 (13.7%)	0.02
1945	967 (16.2%)	314 (16.1%)	385 (16.2%)	18 (18.9%)	250 (16.1%)	
1953	1329 (22.2%)	429 (22.0%)	564 (23.7%)	21 (22.1%)	315 (20.3%)	
1955	1422 (23.8%)	441 (22.6%)	584 (24.5%)	24 (25.3%)	373 (24.0%)	
1957	1492 (24.9%)	478 (24.5%)	590 (24.8%)	22 (23.2%)	402 (25.9%)	
Sex						
Male	2957 (49.5%)	906 (46.4%)	1180 (49.6%)	47 (49.5%)	824 (53.1%)	0.002
Female	3022 (50.5%)	1046 (53.6%)	1199 (50.4%)	48 (50.5%)	729 (46.9%)	
Socioeconomic^b^ status						
Low	193 (3.2%)	74 (3.8%)	55 (2.3%)	5 (5.3%)	59 (3.8%)	0.007
Average	1657 (27.7%)	542 (27.7%)	677 (28.5%)	19 (20.0%)	419 (27.0%)	
High	1543 (25.8%)	513 (26.3%)	588 (24.7%)	22 (23.2%)	420 (27.0%)	
Very high	2401 (40.2%)	763 (39.1%)	1000 (42.0%)	48 (50.5%)	590 (38.0%)	
Missing	185 (3.1%)	60 (3.1%)	59 (2.5%)	1 (1.0%)	65 (4.2%)	

^a^*p* Values were calculated using Chi-square test statistic, comparing participants in the three groups and non-participants, excluding missing.

^b^Based on postal code areas.

### Combined versus FIT-only strategy

**Fig. 2:**
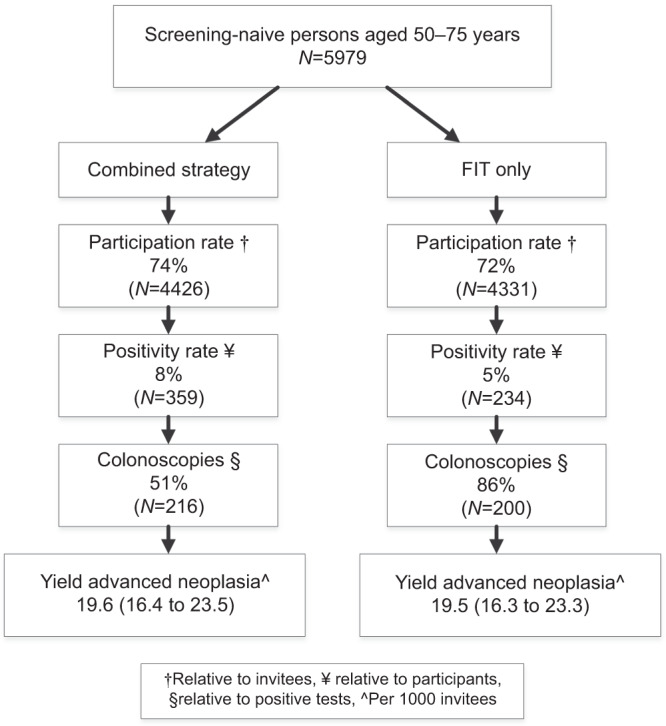
Study enrollment.

For the comparison of the two strategies, we included all 4426 invitees who returned the FIT and the FHQ, FIT only or the FHQ only in the combined strategy (invitations for colonoscopy based on FIT and/or FHQ). Similarly, we included all 4331 invitees who returned the FIT only or the FIT and FHQ in the FIT-only strategy (invitations for colonoscopy based on FIT only). This implies that the participation rate of the combined strategy was marginally but significantly higher (4426/5979; 74.0%) compared to a FIT-only strategy (4331/5979; 72.4%; *p* < 0.001). Figure 2 shows the details of the comparison.

The FHQ was initially found to be positive in 191 (7.7%) of the 2474 participants who had returned the FHQ. After a telephone check, 66 of these (34.6%) appeared to be false positives (Fig. 1). Thirty-three of the 4331 (0.8%) returned FIT results could not be analysed, of whom 9 had also participated in the FHQ. Taking into account the 24 unanalysable tests for the combined strategy and the 33 unanalysable FIT results, the positivity rate for the combined strategy was significantly higher compared to that of the FIT-only strategy: 359 of 4402 (8.2%) versus 234 of 4298 (5.4%; *p* < 0.001).

In 14 individuals, CRC was detected during colonoscopy, all through a positive FIT result. Sixteen colonoscopies were performed in participants with a negative FIT but because of a positive FHQ. One additional advanced adenoma was detected in this group (Table 2). This resulted in a significantly higher positive predictive value for advanced adenomas and neoplasia for the FIT-only strategy compared to the combined strategy.

**Table 2:** Colonoscopy outcomes: combined strategy versus FIT-only strategy.

	Combined strategy (*n*/*N* (%))	FIT-only strategy (*n*/*N* (%))	*p* Value^a^
Detection rate (%)^b^			
CRC	14/216 (6.5%)	14/200 (7.0%)	0.99
Advanced adenoma	103/216 (47.7%)	102/200 (51.0%)	0.56
Advanced neoplasia	117/216 (54.2%)	116/200 (58.0%)	0.49
Non-advanced lesions			
Non-advanced adenoma	49/216 (22.4%)	42/200 (21%)	0.77
SSL+others	21/216 (9.7%)	18/200 (9.0%)	0.93
No lesions	29/216 (13.4%)	24/200 (12.0%)	0.77
Positive predictive value (%)^c^			
CRC	14/359 (4% (2–6%))	14/234 (6% (3–10%))	0.33
Advanced adenoma	103/359 (29% (24–34%))	102/234 (44% (37–50%))	<0.01
Advanced neoplasia	117/359 (33% (28–38%)	116/234 (50% (43–56%))	<0.01

*CRC* colorectal cancer, *SSL* sessile serrated lesion.

^a^*p* Values were calculated using the Chi-square test.

^b^Detection rates were defined as the most advanced lesion per participant relative to all colonoscopies performed.

^c^Positive predictive value was defined as the number of participants with CRC, advanced adenoma or advanced neoplasia relative to the number of positive tests.

Eventually, the yield for AN was 19.6 per 1000 invitees (95% confidence interval (CI): 16.4–23.5) for the combined strategy versus 19.5 per 1000 invitees (95% CI: 16.3–23.3) for FIT-only strategy, a non-significant difference of 0.1 per 1000 invitees (*p* value = 1.0; Fig. 2).

### FHQ results

Of these participants eligible for referral for genetic counselling, 60 were not referred, because they had already received genetic counselling and/or received an advice for surveillance (*n* = 29), because they declined referral (*n* = 30) or were not reached (*n* = 1). Reported reasons for declining referral for genetic counselling were: financial reasons (*n* = 5), other comorbidities (*n* = 4), no perceived added value of counselling (*n* = 7), and no reason mentioned (*n* = 14). Fifty of the 65 (76.9%) participants referred for genetic counselling underwent counselling.

### Genetic testing and counselling

**Fig. 3:**
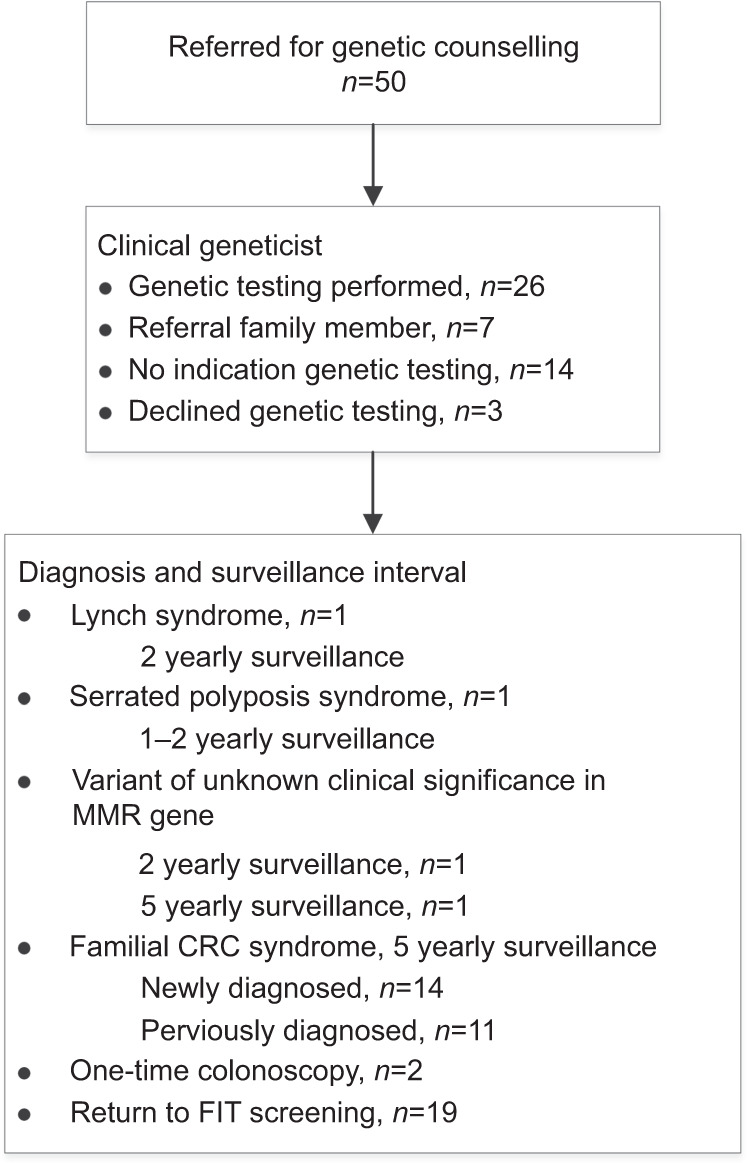
Genetic counselling results.

Of the 50 participants who underwent genetic counselling, 26 underwent genetic testing because of suspicion of Lynch syndrome (Fig. 3). This resulted in the diagnosis of Lynch syndrome (*MSH2* mutation) in one participant and the detection of two participants with gene variants of unknown clinical significance in the *MLH1* gene and the *MSH6* gene, appointed to a 2 yearly and 5 yearly surveillance interval, respectively. Lynch syndrome was excluded by genetic testing in the 23 other participants, of whom 10 were newly diagnosed with familial CRC syndrome, 7 were previously diagnosed with familial CRC syndrome, 2 participants were advised to undergo a one-time colonoscopy and 4 were advised to return to the national population-based FIT screening programme. In seven participants, referral of family members was advised to undergo genetic testing, of whom one was newly diagnosed, one previously diagnosed with familial CRC syndrome and five were advised to return to the national population-based FIT screening programme. In 14 participants, information was requested to exclude Lynch syndrome, of whom 3 were newly diagnosed with familial CRC, 3 were previously diagnosed with familial CRC syndrome and 8 were advised to return to the national population-based FIT screening programme. Three participants had declined genetic testing after genetic counselling, of whom one turned out to have serrated polyposis syndrome at colonoscopy for a newly diagnosed familial CRC syndrome and two were advised to return to the national population-based FIT screening programme.

### Colonoscopy surveillance instead of FIT screening

In addition to the colonoscopies after a positive FIT, 16 colonoscopies were performed as a result of a positive FHQ. Indications for colonoscopy were Lynch syndrome (*n* = 1), a carrier of a variant of unknown clinical significance in the mismatch repair (MMR) gene appointed to a 2-year surveillance interval (*n* = 1), newly diagnosed familial CRC syndrome (*n* = 11), one-time colonoscopy after exclusion of Lynch syndrome (*n* = 1), and previously diagnosed familial CRC syndrome withdrawn from surveillance (*n* = 2). One of the participants who underwent colonoscopy because of a newly diagnosed familial CRC syndrome turned out to have serrated polyposis syndrome. The second participant, carrier of a gene variant of unknown clinical significance in the *MSH6* gene appointed to a 5 yearly surveillance interval, was already undergoing surveillance for familial CRC syndrome. Two patients with a new diagnosis of familial CRC syndrome declined to undergo a colonoscopy and two of them underwent the colonoscopy because of a concurrent positive FIT result. One participant advised to undergo a one-time colonoscopy after exclusion of Lynch syndrome had declined a colonoscopy because of his age.

In the end, we identified 19 individuals who had an indication to undergo a regular surveillance colonoscopy instead of participating in a FIT-based screening programme. These included 1 participant diagnosed with Lynch syndrome, 1 carrier of a variant of unknown clinical significance in the MMR gene appointed to a 2-year surveillance interval, 1 with serrated polyposis syndrome, 14 newly diagnosed individuals with familial CRC syndrome and 2 participants who had withdrawn from surveillance for familial CRC. Fifteen of these 19 (78.9%) participants actually underwent a colonoscopy because of the study and 2 (10.5%) because of a concurrent positive FIT. Two participants had declined surveillance for familial CRC syndrome (10.5%).

## Discussion

In this study, we observed no significant incremental yield in detecting AN after adding a validated online FHQ on family history of CRC and Lynch syndrome-associated tumours to one round of FIT screening. In the almost 6000 invitees, 16 additional colonoscopies were performed for a positive FHQ result, but only 1 additional participant with AN was detected. Despite, we identified 19 individuals who had an indication to undergo colonoscopy surveillance instead of participating in a FIT-based screening programme.

An unfavourable result of this study was the high false positivity rate (35%) using the online FHQ, despite previous validation in patients scheduled for colonoscopy in a private colonoscopy centre.^19^ This prior validation had showed a comparable number of false positives but no false-negative results, when referrals based on questionnaire data were compared with referrals based on data collected in a telephone interview. Therefore, we had integrated the telephone check in the current online FHQ tool and had accepted the risk of having a false-positive result, while adequately detecting all participants at risk. Second, the SES in our sample was slightly higher than that of the national programme, even though the selected regions were considered comparable to the national SES distribution. Nevertheless, the slightly higher SES and addition of the FHQ did not affect the FIT participation rate, FIT positivity rate or AN detection rate.^4^ The higher SES may have influenced participation in the FHQ in a positive way.^26^ We observed that invitees with a lower SES were less likely to return the FHQ. As participants depend on their health insurance for reimbursement of the genetic follow-up examination, and financial reasons were often reported as a reason for declining genetic testing, costs may have been a reason for lower SES invitees not to return the FHQ.

A strength of this study is that it is the first to evaluate the addition of an online FHQ to a national organised FIT-based screening programme assessing the effect in participants with a positive as well as those with negative FIT result. The large randomly selected cohort was screen-naive and consisted of an average-risk population with an age and sex distribution similar to that of the national screening programme. Furthermore, the quality standards of the colonoscopy performed after a positive FHQ were comparable to that of the population screening colonoscopies. Lastly, positive FHQs were verified by a genetic counsellor or clinical geneticist.

In the Netherlands, in the year 2016 the incidence of CRC was 15,306 with a corresponding mortality of 5154 in a population of 16,980,000 individuals.^1^ A previous study in the Dutch population reported that 10% of the affected and 2.3% of the unaffected respondents reported to fulfil the Dutch referral criteria for genetic testing for Lynch syndrome or surveillance for familial CRC syndrome.^27^ These numbers indicate that, in our country, in total almost 400,000 individuals fulfil the criteria for Lynch syndrome or familial CRC syndrome. Although the exact numbers of patients diagnosed with Lynch syndrome and familial CRC syndrome are lacking, the majority of these patients are unaware of their diagnosis and at great risk for developing CRC.^28^ Therefore, the implementation of an FHQ as a screening tool could be beneficial.

Compared with a previously performed study in the Dutch population, the current participation rate of the FHQ was considerably lower.^27^ This could have perhaps been the result of the online nature of the FHQ as predominantly younger invitees with a higher SES had participated in the FHQ. Despite the fact that positivity rates of the FHQ were comparable to previously published studies^7,17,29,30^ and considerable evidence of a higher neoplastic yield among individuals with a family history of CRC, the yield of the combined screening strategy was limited.^31,32^ This may be explained by the low referral rate to the clinical geneticist of only 40% compared to a recently reported uptake of pre-symptomatic genetic testing for Lynch syndrome according to genetic centres of 41–94%.^33^ A large proportion of our patients actively declined referral (24%), did not show up at their appointment or cancelled it (12%). Compliance to appointments is known to be related to perceived urgency of medical clinic visits and associated with a strong recommendation by the physician.^34^ Given the screening situation, participants could have perceived this advice as less urgent and therefore may have declined referral to a clinical geneticist. Lengthy waiting time might also have caused participants to change their minds. Future research should evaluate whether different strategies, for instance without verification by the clinical geneticist, may lead to an increase in diagnostic yield.

Another explanation for the limited diagnostic yield is that a positive family history of CRC considers a life-long risk that is not necessarily associated with an increase in the diagnostic yield in just one single colonoscopy. Future research should investigate whether addition of an FHQ changes the yield after several round of FIT screening.

As in our study only one third of the participants with a positive FHQ result had already received genetic counselling and/or surveillance advice, there seems still room for improvement in the identification of individuals with familial or hereditary CRC syndromes. In the FIT information leaflet, there is no mention of exclusion from participation in FIT screening for patients with a positive family history for CRC. We observed that patients who had already undergone colonoscopy surveillance for a positive family history also participated in the FIT screening programme, indicating that the information for invitees on who should undergo FIT screening could be improved.

In conclusion, we observed no increase in yield after introducing a validated, online FHQ for detecting AN in this study that was embedded in the Dutch FIT-based CRC screening programme. Addition of this FHQ resulted in the identification of a small number of participants at increased risk of developing CRC who should undergo periodic colonoscopy surveillance and not participate in a screening programme based on FIT screening.

## Supplementary information

Supplementary Information Family History Questionnaire in Dutch

Supplementary Information Table 1 Referral criteria for genetic testing and colonoscopy surveillance

## Data Availability

The authors are willing to collaborate in further analyses, on request.
