# Supplementary material for: Addition of an online, validated family history questionnaire to the Dutch FIT-based screening programme did not improve its diagnostic yield
Source: Br J Cancer. 2020 Apr 20;122(12):1865–71. doi: 10.1038/s41416-020-0832-8 (PMC7283285; doi:10.1038/s41416-020-0832-8)
Supplement: Supplementary file 1 — Supplementary Information Family History Questionnaire in Dutch [file 41416_2020_832_MOESM1_ESM.pdf]

BIJLAGE 1: VOORBEELD DIGITALE VRAGENLIJST

Het betreft hier een PDF versie van de digitale vragenlijst. In de werkelijke situatie worden de vragen één voor één gesteld.

Vragenlijst erfelijke darmkanker - Vragen over uzelf

Number	Question	Answers
2.1	Heeft u ooit dikkedarmkanker gehad? Met dikkedarmkanker wordt ook endeldarmkanker bedoeld	○ Ja<br>○ Nee
2.1.1	If 2.1 is equal to 'Ja' answer this question:<br>Hoe oud was u toen dit (voor het eerst) werd ontdekt?	○ Jonger dan 50 jaar<br>○ Tussen 50 en 70 jaar<br>○ Ouder dan 70 jaar
2.1.2	If 2.1 is equal to 'Ja' answer this question:<br>Heeft u vaker dan 1x dikkedarmkanker gehad onder de 70 jaar?	○ Ja<br>○ Nee
2.2	Heeft u behalve dikkedarmkanker ooit een ander soort kanker gehad?	○ Ja<br>○ Nee
2.2.1	If 2.2 is equal to 'Ja' answer this question:<br>Hoe oud was u toen dit werd ontdekt?	○ Jonger dan 50 jaar<br>○ Tussen 50 en 70 jaar<br>○ Ouder dan 70 jaar
2.2.2	If 2.2 is equal to 'Ja' answer this question:<br>In welk orgaan zat deze kanker? U kunt meerdere antwoorden aanvinken	☐ Maag<br>☐ Dunne darm (o.a. twaalfvingerige darm)<br>☐ Alvleesklier<br>☐ Galwegen<br>☐ Nierbekken<br>☐ Nieren<br>☐ Hogere urinewegen<br>☐ Eierstokken<br>☐ Hersenen<br>☐ Talgklier (goedaardig of kwaadaardig)<br>☐ Baarmoeder<br>☐ Baarmoederhals (Hierop wordt getest met het uitstrijkje bij het bevolkingsonderzoek)

- Prostaat
- Borst
- Huid
- Longen
- Lymfeklier/leukemie
- Slokdarm
- Anders
- Onbekend

Vragenlijst erfelijke darmkanker - Erfelijkheidsonderzoek

Number	Question	Answers
3.1	Is er in uw familie ooit erfelijkheidsonderzoek gedaan in verband met darmkanker?	Ja<br>Nee<br>Onbekend
3.1.1	***If 3.1 is equal to 'Ja' answer this question:***<br>Is hierbij een erfelijke aanleg voor darmkanker aangetoond?	Ja<br>Nee<br>Onbekend
3.1.1.1	***If 3.1.1 is equal to 'Ja' answer this question:***<br>Wat is er aangetoond bij het erfelijkheidsonderzoek?	HNPCC/Syndroom van Lynch<br>Familiaire adenomateuze polyposis (FAP)<br>MUTYH geassocieerde polyposis (MAP)<br>Anders<br>Onbekend
3.1.1.2	***If 3.1.1 is equal to 'Ja' answer this question:***<br>Bent u drager?	Ja<br>Nee<br>Onbekend

Vragenlijst erfelijke darmkanker - Vragen over uw moeder

Number	Question	Answers
4.1	Heeft uw moeder ooit dikkedarmkanker gehad?	Ja<br>Nee<br>Onbekend

4.1.1	***If 4.1 is equal to 'Ja' answer this question:***<br>Hoe oud was zij toen dit bij haar werd ontdekt?	◯ Jonger dan 50 jaar<br>◯ Tussen 50 en 70 jaar<br>◯ Ouder dan 70 jaar<br>◯ Onbekend
4.2	Heeft uw moeder ooit een ander soort kanker dan dikkedarmkanker gehad?	◯ Ja<br>◯ Nee<br>◯ Onbekend
4.2.1	***If 4.2 is equal to 'Ja' answer this question:***<br>Hoe oud was zij toen dit bij haar werd ontdekt?	◯ Jonger dan 50 jaar<br>◯ Tussen 50 en 70 jaar<br>◯ Ouder dan 70 jaar<br>◯ Onbekend
4.2.2	***If 4.2 is equal to 'Ja' answer this question:***<br>In welk orgaan zat deze kanker? U kunt meerdere antwoorden aanvinken	☐ Maag<br>☐ Dunne darm (o.a. twaalfvingerige darm)<br>☐ Alvleesklier<br>☐ Galwegen<br>☐ Nierbekken<br>☐ Nieren<br>☐ Hogere urinewegen<br>☐ Eierstokken<br>☐ Hersenen<br>☐ Talgklier (goedaardig of kwaadaardig)<br>☐ Baarmoeder<br>☐ Baarmoederhals (Hierop wordt getest met het uitstrijkje bij het bevolkingsonderzoek)<br>☐ Prostaat<br>☐ Borst<br>☐ Huid<br>☐ Longen<br>☐ Lymfeklier/leukemie<br>☐ Slokdarm<br>☐ Anders<br>☐ Onbekend

Vragenlijst erfelijke darmkanker - Vragen over uw vader

Number	Question	Answers
5.1	Heeft uw vader ooit dikkedarmkanker gehad?	◯ Ja

		Nee<br>Onbekend
5.1.1	***If 5.1 is equal to 'Ja' answer this question:***<br>Hoe oud was hij toen dit bij hem werd ontdekt?	Jonger dan 50 jaar<br>Tussen 50 en 70 jaar<br>Ouder dan 70 jaar<br>Onbekend
5.2	Heeft uw vader ooit een ander soort kanker dan dikkedarmkanker gehad?	Ja<br>Nee<br>Onbekend
5.2.1	***If 5.2 is equal to 'Ja' answer this question:***<br>Hoe oud was hij toen dit bij hem werd ontdekt?	Jonger dan 50 jaar<br>Tussen 50 en 70 jaar<br>Ouder dan 70 jaar<br>Onbekend
5.2.2	***If 5.2 is equal to 'Ja' answer this question:***<br>In welk orgaan zat deze kanker? U kunt meerdere antwoorden aanvinken	Maag<br>Dunne darm (o.a. twaalfvingerige darm)<br>Alvleesklier<br>Galwegen<br>Nierbekken<br>Nieren<br>Hogere urinewegen<br>Eierstokken<br>Hersenen<br>Talgklier (goedaardig of kwaadaardig)<br>Baarmoeder<br>Baarmoederhals (Hierop wordt getest met het uitstrijkje bij het bevolkingsonderzoek)<br>Prostaat<br>Borst<br>Huid<br>Longen<br>Lymfeklier/leukemie<br>Slokdarm<br>Anders<br>Onbekend

Vragenlijst erfelijke darmkanker - Vragen over eventuele broers/zussen

Number	Question	Answers
6.1	Hoeveel broers en/of zussen heeft u? Halfbroers en halfzussen tellen hierbij ook mee	○ 0<br>○ 1<br>○ 2<br>○ 3<br>○ Meer dan 3<br>○ Onbekend
6.1.1	***If 6.1 is bigger than '0' answer this question:***<br>Heeft een broer of zus van u ooit dikkedarmkanker gehad? Mocht u het niet van iedereen weten, vink dan het meest volledige antwoord aan	○ Ja, 1 broer/zus<br>○ Ja, 2 broers/zussen<br>○ Ja, 3 broers/zussen<br>○ Ja, meer dan 3 broers/zussen<br>○ Nee<br>○ Onbekend
6.1.1.1	***If 6.1.1 is bigger than 'Nee' answer this question:***<br>Hoe oud was broer of zus 1 toen dit werd ontdekt?	○ Jonger dan 50 jaar<br>○ Tussen 50 en 70 jaar<br>○ Ouder dan 70 jaar<br>○ Onbekend
6.1.1.2	***If 6.1.1 is bigger than 'Ja, 1 broer/zus' answer this question:***<br>Hoe oud was broer of zus 2 toen dit werd ontdekt?	○ Jonger dan 50 jaar<br>○ Tussen 50 en 70 jaar<br>○ Ouder dan 70 jaar<br>○ Onbekend
6.1.1.3	***If 6.1.1 is bigger than 'Ja, 2 broers/zussen' answer this question:***<br>Hoe oud was broer of zus 3 toen dit werd ontdekt?	○ Jonger dan 50 jaar<br>○ Tussen 50 en 70 jaar<br>○ Ouder dan 70 jaar<br>○ Onbekend
6.1.1.4	***If 6.1.1 is bigger than 'Ja, 3 broers/zussen' answer this question:***<br>Hoe oud was broer of zus 4 toen dit werd ontdekt?	○ Jonger dan 50 jaar<br>○ Tussen 50 en 70 jaar<br>○ Ouder dan 70 jaar<br>○ Onbekend
6.1.2	***If 6.1 is bigger than '0' answer this question:***<br>Heeft een broer of zus van u ooit een ander soort kanker dan dikkedarmkanker gehad? Mocht u het niet van iedereen weten, vink dan het meest volledige antwoord aan	○ Ja, 1 broer/zus<br>○ Ja, 2 broers/zussen<br>○ Ja, 3 broers/zussen<br>○ Ja, meer dan 3 broers/zussen<br>○ Nee<br>○ Onbekend
6.1.2.1	***If 6.1.2 is bigger than 'Nee' answer this question:***	○ Jonger dan 50 jaar

Hoe oud was broer of zus 1 toen dit werd ontdekt?

- [illegible]
- Tussen 50 en 70 jaar
- Ouder dan 70 jaar
- Onbekend

6.1.2.2 ***If 6.1.2 is bigger than 'Nee' answer this question:***
In welk orgaan zat deze kanker? U kunt meerdere antwoorden aanvinken

- Maag
- Dunne darm (o.a. twaalfvingerige darm)
- Alvleesklier
- Galwegen
- Nierbekken
- Nieren
- Hogere urinewegen
- Eierstokken
- Hersenen
- Talgklier (goedaardig of kwaadaardig)
- Baarmoeder
- Baarmoederhals (Hierop wordt getest met het uitstrijkje bij het bevolkingsonderzoek)
- Prostaat
- Borst
- Huid
- Longen
- Lymfeklier/leukemie
- Slokdarm
- Anders
- Onbekend

6.1.2.3 ***If 6.1.2 is bigger than 'Ja, 1 broer/zus' answer this question:***
Hoe oud was broer of zus 2 toen dit werd ontdekt?

- Jonger dan 50 jaar
- Tussen 50 en 70 jaar
- Ouder dan 70 jaar
- Onbekend

6.1.2.4 ***If 6.1.2 is bigger than 'Ja, 1 broer/zus' answer this question:***
In welk orgaan zat deze kanker? U kunt meerdere antwoorden aanvinken

- Maag
- Dunne darm (o.a. twaalfvingerige darm)
- Alvleesklier
- Galwegen
- Nierbekken
- Nieren
- Hogere urinewegen
- Eierstokken
- Hersenen
- Talgklier (goedaardig of kwaadaardig)
- Baarmoeder
- Baarmoederhals (Hierop wordt getest met het uitstrijkje bij het bevolkingsonderzoek)

- Prostaat
- Borst
- Huid
- Longen
- Lymfeklier/leukemie
- Slokdarm
- Anders
- Onbekend

6.1.2.5 ***If 6.1.2 is bigger than 'Ja, 2 broers/zussen' answer this question:***
Hoe oud was broer of zus 3 toen dit werd ontdekt?

- Jonger dan 50 jaar
- Tussen 50 en 70 jaar
- Ouder dan 70 jaar
- Onbekend

6.1.2.6 ***If 6.1.2 is bigger than 'Ja, 2 broers/zussen' answer this question:***
In welk orgaan zat deze kanker? U kunt meerdere antwoorden aanvinken

- Maag
- Dunne darm (o.a. twaalfvingerige darm)
- Alvleesklier
- Galwegen
- Nierbekken
- Nieren
- Hogere urinewegen
- Eierstokken
- Hersenen
- Talgklier (goedaardig of kwaadaardig)
- Baarmoeder
- Baarmoederhals (Hierop wordt getest met het uitstrijkje bij het bevolkingsonderzoek)
- Prostaat
- Borst
- Huid
- Longen
- Lymfeklier/leukemie
- Slokdarm
- Anders
- Onbekend

6.1.2.7 ***If 6.1.2 is bigger than 'Ja, 3 broers/zussen' answer this question:***
Hoe oud was broer of zus 4 toen dit werd ontdekt?

- Jonger dan 50 jaar
- Tussen 50 en 70 jaar
- Ouder dan 70 jaar
- Onbekend

6.1.2.8 ***If 6.1.2 is bigger than 'Ja, 3 broers/zussen' answer this question:***
In welk orgaan zat deze kanker? U kunt meerdere antwoorden aanvinken

- Maag
- Dunne darm (o.a. twaalfvingerige darm)
- Alvleesklier

- ☐ Galwegen
- ☐ Nierbekken
- ☐ Nieren
- ☐ Hogere urinewegen
- ☐ Eierstokken
- ☐ Hersenen
- ☐ Talgklier (goedaardig of kwaadaardig)
- ☐ Baarmoeder
- ☐ Baarmoederhals (Hierop wordt getest met het uitstrijkje bij het bevolkingsonderzoek)
- ☐ Prostaat
- ☐ Borst
- ☐ Huid
- ☐ Longen
- ☐ Lymfeklier/leukemie
- ☐ Slokdarm
- ☐ Anders
- ☐ Onbekend

Vragenlijst erfelijke darmkanker - Vragen over eventuele kinderen

Number	Question	Answers
7.1	Hoeveel kinderen heeft u?	○ 0<br>○ 1<br>○ 2<br>○ 3<br>○ Meer dan 3<br>○ Onbekend
7.1.1	***If 7.1 is bigger than '0' answer this question:***<br>Heeft een kind van u ooit dikkedarmkanker gehad? Mocht u het niet van iedereen weten, vink dan het meest volledige antwoord aan	○ Ja, 1 kind<br>○ Ja, 2 kinderen<br>○ Ja, 3 kinderen<br>○ Ja, meer dan 3 kinderen<br>○ Nee<br>○ Onbekend
7.1.1.1	***If 7.1.1 is bigger than 'Nee' answer this question:***<br>Hoe oud was kind 1 toen dit werd ontdekt?	○ Jonger dan 50 jaar<br>○ Tussen 50 en 70 jaar<br>○ Ouder dan 70 jaar<br>○ [illegible]

Onbekend

7.1.1.2	***If 7.1.1 is bigger than 'Ja, 1 kind' answer this question:*** Hoe oud was kind 2 toen dit werd ontdekt?	Jonger dan 50 jaar<br>Tussen 50 en 70 jaar<br>Ouder dan 70 jaar<br>Onbekend
7.1.1.3	***If 7.1.1 is bigger than 'Ja, 2 kinderen' answer this question:*** Hoe oud was kind 3 toen dit werd ontdekt?	Jonger dan 50 jaar<br>Tussen 50 en 70 jaar<br>Ouder dan 70 jaar<br>Onbekend
7.1.1.4	***If 7.1.1 is bigger than 'Ja, 3 kinderen' answer this question:*** Hoe oud was kind 4 toen dit werd ontdekt?	Jonger dan 50 jaar<br>Tussen 50 en 70 jaar<br>Ouder dan 70 jaar<br>Onbekend
7.1.2	***If 7.1 is bigger than '0' answer this question:*** Heeft een kind van u ooit een ander soort kanker dan dikkedarmkanker gehad? Mocht u het niet van iedereen weten, vink dan het meest volledige antwoord aan	Ja, 1 kind<br>Ja, 2 kinderen<br>Ja, 3 kinderen<br>Ja, meer dan 3 kinderen<br>Nee<br>Onbekend
7.1.2.1	***If 7.1.2 is bigger than 'Nee' answer this question:*** Hoe oud was kind 1 toen dit werd ontdekt?	Jonger dan 50 jaar<br>Tussen 50 en 70 jaar<br>Ouder dan 70 jaar<br>Onbekend
7.1.2.2	***If 7.1.2 is bigger than 'Nee' answer this question:*** In welk orgaan zat deze kanker? U kunt meerdere antwoorden aanvinken	Maag<br>Dunne darm (o.a. twaalfvingerige darm)<br>Alvleesklier<br>Galwegen<br>Nierbekken<br>Nieren<br>Hogere urinewegen<br>Eierstokken<br>Hersenen<br>Talgklier (goedaardig of kwaadaardig)<br>Baarmoeder<br>Baarmoederhals (Hierop wordt getest met het uitstrijkje bij het bevolkingsonderzoek)<br>Prostaat<br>Borst<br>Huid

- Huid
- Longen
- Lymfeklier/leukemie
- Slokdarm
- Anders
- Onbekend

7.1.2.3 ***If 7.1.2 is bigger than 'Ja, 1 kind' answer this question:***
Hoe oud was kind 2 toen dit werd ontdekt?

- Jonger dan 50 jaar
- Tussen 50 en 70 jaar
- Ouder dan 70 jaar
- Onbekend

7.1.2.4 ***If 7.1.2 is bigger than 'Ja, 1 kind' answer this question:***
In welk orgaan zat deze kanker? U kunt meerdere antwoorden aanvinken

- Maag
- Dunne darm (o.a. twaalfvingerige darm)
- Alvleesklier
- Galwegen
- Nierbekken
- Nieren
- Hogere urinewegen
- Eierstokken
- Hersenen
- Talgklier (goedaardig of kwaadaardig)
- Baarmoeder
- Baarmoederhals (Hierop wordt getest met het uitstrijkje bij het bevolkingsonderzoek)
- Prostaat
- Borst
- Huid
- Longen
- Lymfeklier/leukemie
- Slokdarm
- Anders
- Onbekend

7.1.2.5 ***If 7.1.2 is bigger than 'Ja, 2 kinderen' answer this question:***
Hoe oud was kind 3 toen dit werd ontdekt?

- Jonger dan 50 jaar
- Tussen 50 en 70 jaar
- Ouder dan 70 jaar
- Onbekend

7.1.2.6 ***If 7.1.2 is bigger than 'Ja, 2 kinderen' answer this question:***
In welk orgaan zat deze kanker? U kunt meerdere antwoorden aanvinken

- Maag
- Dunne darm (o.a. twaalfvingerige darm)
- Alvleesklier
- Galwegen
- Nierbekken
- Nieren
- Hogere urinewegen

- Hogere urinewegen
- Eierstokken
- Hersenen
- Talgklier (goedaardig of kwaadaardig)
- Baarmoeder
- Baarmoederhals (Hierop wordt getest met het uitstrijkje bij het bevolkingsonderzoek)
- Prostaat
- Borst
- Huid
- Longen
- Lymfeklier/leukemie
- Slokdarm
- Anders
- Onbekend

7.1.2.7 ***If 7.1.2 is bigger than 'Ja, 3 kinderen' answer this question:***
Hoe oud was kind 4 toen dit werd ontdekt?

- Jonger dan 50 jaar
- Tussen 50 en 70 jaar
- Ouder dan 70 jaar
- Onbekend

7.1.2.8 ***If 7.1.2 is bigger than 'Ja, 3 kinderen' answer this question:***
In welk orgaan zat deze kanker? U kunt meerdere antwoorden aanvinken

- Maag
- Dunne darm (o.a. twaalfvingerige darm)
- Alvleesklier
- Galwegen
- Nierbekken
- Nieren
- Hogere urinewegen
- Eierstokken
- Hersenen
- Talgklier (goedaardig of kwaadaardig)
- Baarmoeder
- Baarmoederhals (Hierop wordt getest met het uitstrijkje bij het bevolkingsonderzoek)
- Prostaat
- Borst
- Huid
- Longen
- Lymfeklier/leukemie
- Slokdarm
- Anders
- Onbekend

Vragenlijst erfelijke darmkanker - Vragen over grootouders

Number	Question	Answers
8.1	Heeft een van uw grootouders ooit dikkedarmkanker gehad? Met grootouders worden de vader en moeder van uw eigen ouders bedoeld. Mocht u het niet van iedereen weten, vink dan het meest volledige antwoord aan	Ja, 1 grootouder<br>Ja, 2 grootouders<br>Ja, 3 grootouders<br>Ja, 4 grootouders<br>Nee<br>Onbekend
8.1.1	***If 8.1 is bigger than 'Nee' answer this question:***<br>Hoe oud was grootouder 1 toen dit werd ontdekt?	Jonger dan 50 jaar<br>Tussen 50 en 70 jaar<br>Ouder dan 70 jaar<br>Onbekend
8.1.2	***If 8.1 is bigger than 'Nee' answer this question:***<br>Kruis aan om wie het hierbij ging:	De vader van uw vader<br>De moeder van uw vader<br>De vader van uw moeder<br>De moeder van uw moeder
8.1.3	***If 8.1 is bigger than 'Ja, 1 grootouder' answer this question:***<br>Hoe oud was grootouder 2 toen dit werd ontdekt?	Jonger dan 50 jaar<br>Tussen 50 en 70 jaar<br>Ouder dan 70 jaar<br>Onbekend
8.1.4	***If 8.1 is bigger than 'Ja, 1 grootouder' answer this question:***<br>Kruis aan om wie het hierbij ging:	De vader van uw vader<br>De moeder van uw vader<br>De vader van uw moeder<br>De moeder van uw moeder
8.1.5	***If 8.1 is bigger than 'Ja, 2 grootouders' answer this question:***<br>Hoe oud was grootouder 3 toen dit werd ontdekt?	Jonger dan 50 jaar<br>Tussen 50 en 70 jaar<br>Ouder dan 70 jaar<br>Onbekend
8.1.6	***If 8.1 is bigger than 'Ja, 2 grootouders' answer this question:***<br>Kruis aan om wie het hierbij ging:	De vader van uw vader<br>De moeder van uw vader<br>De vader van uw moeder<br>De moeder van uw moeder
8.1.7	***If 8.1 is bigger than 'Ja, 3 grootouders' answer this question:***	Jonger dan 50 jaar<br>Tussen 50 en 70 jaar

	Hoe oud was grootouder 4 toen dit werd ontdekt?	◯ Ouder dan 70 jaar<br>◯ Onbekend
8.1.8	***If 8.1 is bigger than 'Ja, 3 grootouders' answer this question:***<br>Kruis aan om wie het hierbij ging:	◯ De vader van uw vader<br>◯ De moeder van uw vader<br>◯ De vader van uw moeder<br>◯ De moeder van uw moeder
8.2	Heeft een van uw grootouders ooit een ander soort kanker dan dikkedarmkanker gehad? Mocht u het niet van iedereen weten, vink dan het meest volledige antwoord aan	◯ Ja, 1 grootouder<br>◯ Ja, 2 grootouders<br>◯ Ja, 3 grootouders<br>◯ Ja, 4 grootouders<br>◯ Nee<br>◯ Onbekend
8.2.1	***If 8.2 is bigger than 'Nee' answer this question:***<br>Hoe oud was grootouder 1 toen dit werd ontdekt?	◯ Jonger dan 50 jaar<br>◯ Tussen 50 en 70 jaar<br>◯ Ouder dan 70 jaar<br>◯ Onbekend
8.2.2	***If 8.2 is bigger than 'Nee' answer this question:***<br>Kruis aan om wie het hierbij ging:	◯ De vader van uw vader<br>◯ De moeder van uw vader<br>◯ De vader van uw moeder<br>◯ De moeder van uw moeder
8.2.3	***If 8.2 is bigger than 'Nee' answer this question:***<br>In welk orgaan zat deze kanker? U kunt meerdere antwoorden aanvinken	☐ Maag<br>☐ Dunne darm (o.a. twaalfvingerige darm)<br>☐ Alvleesklier<br>☐ Galwegen<br>☐ Nierbekken<br>☐ Nieren<br>☐ Hogere urinewegen<br>☐ Eierstokken<br>☐ Hersenen<br>☐ Talgklier (goedaardig of kwaadaardig)<br>☐ Baarmoeder<br>☐ Baarmoederhals (Hierop wordt getest met het uitstrijkje bij het bevolkingsonderzoek)<br>☐ Prostaat<br>☐ Borst<br>☐ Huid<br>☐ Longen<br>☐ Lymfeklier/leukemie<br>☐ Slokdarm

- Anders
- Onbekend

8.2.4	***If 8.2 is bigger than 'Ja, 1 grootouder' answer this question:*** Hoe oud was grootouder 2 toen dit werd ontdekt?	Jonger dan 50 jaar<br>Tussen 50 en 70 jaar<br>Ouder dan 70 jaar<br>Onbekend
8.2.5	***If 8.2 is bigger than 'Ja, 1 grootouder' answer this question:*** Kruis aan om wie het hierbij ging:	De vader van uw vader<br>De moeder van uw vader<br>De vader van uw moeder<br>De moeder van uw moeder
8.2.6	***If 8.2 is bigger than 'Ja, 1 grootouder' answer this question:*** In welk orgaan zat deze kanker? U kunt meerdere antwoorden aanvinken	Maag<br>Dunne darm (o.a. twaalfvingerige darm)<br>Alvleesklier<br>Galwegen<br>Nierbekken<br>Nieren<br>Hogere urinewegen<br>Eierstokken<br>Hersenen<br>Talgklier (goedaardig of kwaadaardig)<br>Baarmoeder<br>Baarmoederhals (Hierop wordt getest met het uitstrijkje bij het bevolkingsonderzoek)<br>Prostaat<br>Borst<br>Huid<br>Longen<br>Lymfeklier/leukemie<br>Slokdarm<br>Anders<br>Onbekend
8.2.7	***If 8.2 is bigger than 'Ja, 2 grootouders' answer this question:*** Hoe oud was grootouder 3 toen dit werd ontdekt?	Jonger dan 50 jaar<br>Tussen 50 en 70 jaar<br>Ouder dan 70 jaar<br>Onbekend
8.2.8	***If 8.2 is bigger than 'Ja, 2 grootouders' answer this question:*** Kruis aan om wie het hierbij ging:	De vader van uw vader<br>De moeder van uw vader<br>De vader van uw moeder<br>De moeder van uw moeder

8.2.9 ***If 8.2 is bigger than 'Ja, 2 grootouders' answer this question:***
In welk orgaan zat deze kanker? U kunt meerdere antwoorden aanvinken

- ☐ Maag
- ☐ Dunne darm (o.a. twaalfvingerige darm)
- ☐ Alvleesklier
- ☐ Galwegen
- ☐ Nierbekken
- ☐ Nieren
- ☐ Hogere urinewegen
- ☐ Eierstokken
- ☐ Hersenen
- ☐ Talgklier (goedaardig of kwaadaardig)
- ☐ Baarmoeder
- ☐ Baarmoederhals (Hierop wordt getest met het uitstrijkje bij het bevolkingsonderzoek)
- ☐ Prostaat
- ☐ Borst
- ☐ Huid
- ☐ Longen
- ☐ Lymfeklier/leukemie
- ☐ Slokdarm
- ☐ Anders
- ☐ Onbekend

8.2.10 ***If 8.2 is bigger than 'Ja, 3 grootouders' answer this question:***
Hoe oud was grootouder 4 toen dit werd ontdekt?

- ○ Jonger dan 50 jaar
- ○ Tussen 50 en 70 jaar
- ○ Ouder dan 70 jaar
- ○ Onbekend

8.2.11 ***If 8.2 is bigger than 'Ja, 3 grootouders' answer this question:***
Kruis aan om wie het hierbij ging:

- ○ De vader van uw vader
- ○ De moeder van uw vader
- ○ De vader van uw moeder
- ○ De moeder van uw moeder

8.2.12 ***If 8.2 is bigger than 'Ja, 3 grootouders' answer this question:***
In welk orgaan zat deze kanker? U kunt meerdere antwoorden aanvinken

- ☐ Maag
- ☐ Dunne darm (o.a. twaalfvingerige darm)
- ☐ Alvleesklier
- ☐ Galwegen
- ☐ Nierbekken
- ☐ Nieren
- ☐ Hogere urinewegen
- ☐ Eierstokken
- ☐ Hersenen
- ☐ Talgklier (goedaardig of kwaadaardig)
- ☐ Baarmoeder
- ☐ Baarmoederhals (Hierop wordt getest

		met het uitstrijkje bij het bevolkingsonderzoek)<br>☐ Prostaat<br>☐ Borst<br>☐ Huid<br>☐ Longen<br>☐ Lymfeklier/leukemie<br>☐ Slokdarm<br>☐ Anders<br>☐ Onbekend

Vragenlijst erfelijke darmkanker - Vragen over eventuele kinderen van broers/zussen

Number	Question	Answers
9.1	***If 6.1 is bigger than '0' answer this question:***<br>Heeft een van uw broers en/of zussen kinderen (ook degenen die overleden zijn)? Dit zijn dus uw neven en nichten	○ Ja<br>○ Nee<br>○ Onbekend
9.1.1	***If 9.1 is equal to 'Ja' answer this question:***<br>Heeft een kind van uw broers en/of zussen ooit dikkedarmkanker gehad? Mocht u het niet van iedereen weten, vink dan het meest volledige antwoord aan	○ Ja, 1 kind<br>○ Ja, 2 kinderen<br>○ Ja, 3 kinderen<br>○ Ja, meer dan 3 kinderen<br>○ Nee<br>○ Onbekend
9.1.1.1	***If 9.1.1 is bigger than 'Nee' answer this question:***<br>Hoe oud was kind 1 toen dit werd ontdekt?	○ Jonger dan 50 jaar<br>○ Tussen 50 en 70 jaar<br>○ Ouder dan 70 jaar<br>○ Onbekend
9.1.1.2	***If 9.1.1 is bigger than 'Ja, 1 kind' answer this question:***<br>Hoe oud was kind 2 toen dit werd ontdekt?	○ Jonger dan 50 jaar<br>○ Tussen 50 en 70 jaar<br>○ Ouder dan 70 jaar<br>○ Onbekend
9.1.1.3	***If 9.1.1 is bigger than 'Ja, 2 kinderen' answer this question:***<br>Hoe oud was kind 3 toen dit werd ontdekt?	○ Jonger dan 50 jaar<br>○ Tussen 50 en 70 jaar<br>○ Ouder dan 70 jaar<br>○ Onbekend

9.1.1.4	***If 9.1.1 is bigger than 'Ja, 3 kinderen' answer this question:***<br>Hoe oud was kind 4 toen dit werd ontdekt?	○ Jonger dan 50 jaar<br>○ Tussen 50 en 70 jaar<br>○ Ouder dan 70 jaar<br>○ Onbekend
9.1.2	***If 9.1 is equal to 'Ja' answer this question:***<br>Heeft een kind van uw broers en/of zussen ooit een ander soort kanker dan dikkedarmkanker gehad? Mocht u het niet van iedereen weten, vink dan het meest volledige antwoord aan	○ Ja, 1 kind<br>○ Ja, 2 kinderen<br>○ Ja, 3 kinderen<br>○ Ja, meer dan 3 kinderen<br>○ Nee<br>○ Onbekend
9.1.2.1	***If 9.1.2 is bigger than 'Nee' answer this question:***<br>Hoe oud was kind 1 toen dit werd ontdekt?	○ Jonger dan 50 jaar<br>○ Tussen 50 en 70 jaar<br>○ Ouder dan 70 jaar<br>○ Onbekend
9.1.2.2	***If 9.1.2 is bigger than 'Nee' answer this question:***<br>In welk orgaan zat deze kanker? U kunt meerdere antwoorden aanvinken	☐ Maag<br>☐ Dunne darm (o.a. twaalfvingerige darm)<br>☐ Alvleesklier<br>☐ Galwegen<br>☐ Nierbekken<br>☐ Nieren<br>☐ Hogere urinewegen<br>☐ Eierstokken<br>☐ Hersenen<br>☐ Talgklier (goedaardig of kwaadaardig)<br>☐ Baarmoeder<br>☐ Baarmoederhals (Hierop wordt getest met het uitstrijkje bij het bevolkingsonderzoek)<br>☐ Prostaat<br>☐ Borst<br>☐ Huid<br>☐ Longen<br>☐ Lymfeklier/leukemie<br>☐ Slokdarm<br>☐ Anders<br>☐ Onbekend
9.1.2.3	***If 9.1.2 is bigger than 'Ja, 1 kind' answer this question:***<br>Hoe oud was kind 2 toen dit werd ontdekt?	○ Jonger dan 50 jaar<br>○ Tussen 50 en 70 jaar<br>○ Ouder dan 70 jaar<br>○ Onbekend
[illegible]	[illegible]	

9.1.2.4 ***If 9.1.2 is bigger than 'Ja, 1 kind' answer this question:***
In welk orgaan zat deze kanker? U kunt meerdere antwoorden aanvinken

- ☐ Maag
- ☐ Dunne darm (o.a. twaalfvingerige darm)
- ☐ Alvleesklier
- ☐ Galwegen
- ☐ Nierbekken
- ☐ Nieren
- ☐ Hogere urinewegen
- ☐ Eierstokken
- ☐ Hersenen
- ☐ Talgklier (goedaardig of kwaadaardig)
- ☐ Baarmoeder
- ☐ Baarmoederhals (Hierop wordt getest met het uitstrijkje bij het bevolkingsonderzoek)
- ☐ Prostaat
- ☐ Borst
- ☐ Huid
- ☐ Longen
- ☐ Lymfeklier/leukemie
- ☐ Slokdarm
- ☐ Anders
- ☐ Onbekend

9.1.2.5 ***If 9.1.2 is bigger than 'Ja, 2 kinderen' answer this question:***
Hoe oud was kind 3 toen dit werd ontdekt?

- ○ Jonger dan 50 jaar
- ○ Tussen 50 en 70 jaar
- ○ Ouder dan 70 jaar
- ○ Onbekend

9.1.2.6 ***If 9.1.2 is bigger than 'Ja, 2 kinderen' answer this question:***
In welk orgaan zat deze kanker? U kunt meerdere antwoorden aanvinken

- ☐ Maag
- ☐ Dunne darm (o.a. twaalfvingerige darm)
- ☐ Alvleesklier
- ☐ Galwegen
- ☐ Nierbekken
- ☐ Nieren
- ☐ Hogere urinewegen
- ☐ Eierstokken
- ☐ Hersenen
- ☐ Talgklier (goedaardig of kwaadaardig)
- ☐ Baarmoeder
- ☐ Baarmoederhals (Hierop wordt getest met het uitstrijkje bij het bevolkingsonderzoek)
- ☐ Prostaat
- ☐ Borst
- ☐ Huid
- ☐ Longen
- ☐ Lymfeklier/leukemie

		Lymfeklier/leukemie<br>Slokdarm<br>Anders<br>Onbekend
9.1.2.7	***If 9.1.2 is bigger than 'Ja, 3 kinderen' answer this question:***<br>Hoe oud was kind 4 toen dit werd ontdekt?	Jonger dan 50 jaar<br>Tussen 50 en 70 jaar<br>Ouder dan 70 jaar<br>Onbekend
9.1.2.8	***If 9.1.2 is bigger than 'Ja, 3 kinderen' answer this question:***<br>In welk orgaan zat deze kanker? U kunt meerdere antwoorden aanvinken	Maag<br>Dunne darm (o.a. twaalfvingerige darm)<br>Alvleesklier<br>Galwegen<br>Nierbekken<br>Nieren<br>Hogere urinewegen<br>Eierstokken<br>Hersenen<br>Talgklier (goedaardig of kwaadaardig)<br>Baarmoeder<br>Baarmoederhals (Hierop wordt getest met het uitstrijkje bij het bevolkingsonderzoek)<br>Prostaat<br>Borst<br>Huid<br>Longen<br>Lymfeklier/leukemie<br>Slokdarm<br>Anders<br>Onbekend

Vragenlijst erfelijke darmkanker - Vragen over eventuele broers/zussen van moeder

Number	Question	Answers
10.1	Hoeveel broers en/of zussen heeft uw moeder (ook degenen die overleden zijn)? Dit zijn dus uw ooms en/of tantes	0<br>1<br>2<br>3<br>Meer dan 3

		[illegible]<br>◯ Onbekend
10.1.1	***If 10.1 is bigger than '0' answer this question:***<br>Heeft een broer of zus van uw moeder ooit dikkedarmkanker gehad? Mocht u het niet van iedereen weten, vink dan het meest volledige antwoord aan	◯ Ja, 1 broer/zus<br>◯ Ja, 2 broers/zussen<br>◯ Ja, 3 broers/zussen<br>◯ Ja, meer dan 3 broers/zussen<br>◯ Nee<br>◯ Onbekend
10.1.1.1	***If 10.1.1 is bigger than 'Nee' answer this question:***<br>Hoe oud was oom of tante 1 toen dit werd ontdekt?	◯ Jonger dan 50 jaar<br>◯ Tussen 50 en 70 jaar<br>◯ Ouder dan 70 jaar<br>◯ Onbekend
10.1.1.2	***If 10.1.1 is bigger than 'Ja, 1 broer/zus' answer this question:***<br>Hoe oud was oom of tante 2 toen dit werd ontdekt?	◯ Jonger dan 50 jaar<br>◯ Tussen 50 en 70 jaar<br>◯ Ouder dan 70 jaar<br>◯ Onbekend
10.1.1.3	***If 10.1.1 is bigger than 'Ja, 2 broers/zussen' answer this question:***<br>Hoe oud was oom of tante 3 toen dit werd ontdekt?	◯ Jonger dan 50 jaar<br>◯ Tussen 50 en 70 jaar<br>◯ Ouder dan 70 jaar<br>◯ Onbekend
10.1.1.4	***If 10.1.1 is bigger than 'Ja, 3 broers/zussen' answer this question:***<br>Hoe oud was oom of tante 4 toen dit werd ontdekt?	◯ Jonger dan 50 jaar<br>◯ Tussen 50 en 70 jaar<br>◯ Ouder dan 70 jaar<br>◯ Onbekend
10.1.2	***If 10.1 is bigger than '0' answer this question:***<br>Heeft een broer of zus van uw moeder ooit een ander soort kanker dan dikkedarmkanker gehad? Mocht u het niet van iedereen weten, vink dan het meest volledige antwoord aan	◯ Ja, 1 broer/zus<br>◯ Ja, 2 broers/zussen<br>◯ Ja, 3 broers/zussen<br>◯ Ja, meer dan 3 broers/zussen<br>◯ Nee<br>◯ Onbekend
10.1.2.1	***If 10.1.2 is bigger than 'Nee' answer this question:***<br>Hoe oud was oom of tante 1 toen dit werd ontdekt?	◯ Jonger dan 50 jaar<br>◯ Tussen 50 en 70 jaar<br>◯ Ouder dan 70 jaar<br>◯ Onbekend
10.1.2.2	***If 10.1.2 is bigger than 'Nee' answer this question:***<br>In welk orgaan zat deze kanker? U kunt meerdere antwoorden aanvinken	☐ Maag<br>☐ Dunne darm (o.a. twaalfvingerige darm)<br>☐ Alvleesklier

- Galwegen
- Nierbekken
- Nieren
- Hogere urinewegen
- Eierstokken
- Hersenen
- Talgklier (goedaardig of kwaadaardig)
- Baarmoeder
- Baarmoederhals (Hierop wordt getest met het uitstrijkje bij het bevolkingsonderzoek)
- Prostaat
- Borst
- Huid
- Longen
- Lymfeklier/leukemie
- Slokdarm
- Anders
- Onbekend

10.1.2.3 ***If 10.1.2 is bigger than 'Ja, 1 broer/zus' answer this question:***
Hoe oud was oom of tante 2 toen dit werd ontdekt?

- Jonger dan 50 jaar
- Tussen 50 en 70 jaar
- Ouder dan 70 jaar
- Onbekend

10.1.2.4 ***If 10.1.2 is bigger than 'Ja, 1 broer/zus' answer this question:***
In welk orgaan zat deze kanker? U kunt meerdere antwoorden aanvinken

- Maag
- Dunne darm (o.a. twaalfvingerige darm)
- Alvleesklier
- Galwegen
- Nierbekken
- Nieren
- Hogere urinewegen
- Eierstokken
- Hersenen
- Talgklier (goedaardig of kwaadaardig)
- Baarmoeder
- Baarmoederhals (Hierop wordt getest met het uitstrijkje bij het bevolkingsonderzoek)
- Prostaat
- Borst
- Huid
- Longen
- Lymfeklier/leukemie
- Slokdarm
- Anders
- Onbekend

Onbekend

10.1.2.5 ***If 10.1.2 is bigger than 'Ja, 2 broers/zussen' answer this question:***
Hoe oud was oom of tante 3 toen dit werd ontdekt?

- Jonger dan 50 jaar
- Tussen 50 en 70 jaar
- Ouder dan 70 jaar
- Onbekend

10.1.2.6 ***If 10.1.2 is bigger than 'Ja, 2 broers/zussen' answer this question:***
In welk orgaan zat deze kanker? U kunt meerdere antwoorden aanvinken

- Maag
- Dunne darm (o.a. twaalfvingerige darm)
- Alvleesklier
- Galwegen
- Nierbekken
- Nieren
- Hogere urinewegen
- Eierstokken
- Hersenen
- Talgklier (goedaardig of kwaadaardig)
- Baarmoeder
- Baarmoederhals (Hierop wordt getest met het uitstrijkje bij het bevolkingsonderzoek)
- Prostaat
- Borst
- Huid
- Longen
- Lymfeklier/leukemie
- Slokdarm
- Anders
- Onbekend

10.1.2.7 ***If 10.1.2 is bigger than 'Ja, 3 broers/zussen' answer this question:***
Hoe oud was oom of tante 4 toen dit werd ontdekt?

- Jonger dan 50 jaar
- Tussen 50 en 70 jaar
- Ouder dan 70 jaar
- Onbekend

10.1.2.8 ***If 10.1.2 is bigger than 'Ja, 3 broers/zussen' answer this question:***
In welk orgaan zat deze kanker? U kunt meerdere antwoorden aanvinken

- Maag
- Dunne darm (o.a. twaalfvingerige darm)
- Alvleesklier
- Galwegen
- Nierbekken
- Nieren
- Hogere urinewegen
- Eierstokken
- Hersenen
- Talgklier (goedaardig of kwaadaardig)
- Baarmoeder

		☐ Baarmoederhals (Hierop wordt getest met het uitstrijkje bij het bevolkingsonderzoek)<br>☐ Prostaat<br>☐ Borst<br>☐ Huid<br>☐ Longen<br>☐ Lymfeklier/leukemie<br>☐ Slokdarm<br>☐ Anders<br>☐ Onbekend

Vragenlijst erfelijke darmkanker - Vragen over eventuele broers/zussen van vader

Number	Question	Answers
11.1	Hoeveel broers en/of zussen heeft uw vader (ook degenen die overleden zijn)? Dit zijn dus uw ooms en/of tantes	○ 0<br>○ 1<br>○ 2<br>○ 3<br>○ Meer dan 3<br>○ Onbekend
11.1.1	***If 11.1 is bigger than '0' answer this question:***<br>Heeft een broer of zus van uw vader ooit dikkedarmkanker gehad? Mocht u het niet van iedereen weten, vink dan het meest volledige antwoord aan	○ Ja, 1 broer/zus<br>○ Ja, 2 broers/zussen<br>○ Ja, 3 broers/zussen<br>○ Ja, meer dan 3 broers/zussen<br>○ Nee<br>○ Onbekend
11.1.1.1	***If 11.1.1 is bigger than 'Nee' answer this question:***<br>Hoe oud was oom of tante 1 toen dit werd ontdekt?	○ Jonger dan 50 jaar<br>○ Tussen 50 en 70 jaar<br>○ Ouder dan 70 jaar<br>○ Onbekend
11.1.1.2	***If 11.1.1 is bigger than 'Ja, 1 broer/zus' answer this question:***<br>Hoe oud was oom of tante 2 toen dit werd ontdekt?	○ Jonger dan 50 jaar<br>○ Tussen 50 en 70 jaar<br>○ Ouder dan 70 jaar<br>○ Onbekend
11.1.1.3	***If 11.1.1 is bigger than 'Ja, 2 broers/zussen' answer this question:***	○ Jonger dan 50 jaar

	this question: Hoe oud was oom of tante 3 toen dit werd ontdekt?	Tussen 50 en 70 jaar<br>Ouder dan 70 jaar<br>Onbekend
11.1.1.4	***If 11.1.1 is bigger than 'Ja, 3 broers/zussen' answer this question:*** Hoe oud was oom of tante 4 toen dit werd ontdekt?	Jonger dan 50 jaar<br>Tussen 50 en 70 jaar<br>Ouder dan 70 jaar<br>Onbekend
11.1.2	***If 11.1 is bigger than '0' answer this question:*** Heeft een broer of zus van uw vader ooit een ander soort kanker dan dikkedarmkanker gehad? Mocht u het niet van iedereen weten, vink dan het meest volledige antwoord aan	Ja, 1 broer/zus<br>Ja, 2 broers/zussen<br>Ja, 3 broers/zussen<br>Ja, meer dan 3 broers/zussen<br>Nee<br>Onbekend
11.1.2.1	***If 11.1.2 is bigger than 'Nee' answer this question:*** Hoe oud was oom of tante 1 toen dit werd ontdekt?	Jonger dan 50 jaar<br>Tussen 50 en 70 jaar<br>Ouder dan 70 jaar<br>Onbekend
11.1.2.2	***If 11.1.2 is bigger than 'Nee' answer this question:*** In welk orgaan zat deze kanker? U kunt meerdere antwoorden aanvinken	Maag<br>Dunne darm (o.a. twaalfvingerige darm)<br>Alvleesklier<br>Galwegen<br>Nierbekken<br>Nieren<br>Hogere urinewegen<br>Eierstokken<br>Hersenen<br>Talgklier (goedaardig of kwaadaardig)<br>Baarmoeder<br>Baarmoederhals (Hierop wordt getest met het uitstrijkje bij het bevolkingsonderzoek)<br>Prostaat<br>Borst<br>Huid<br>Longen<br>Lymfeklier/leukemie<br>Slokdarm<br>Anders<br>Onbekend
11.1.2.3	***If 11.1.2 is bigger than 'Ja, 1 broer/zus' answer this question:***	Jonger dan 50 jaar

	question:<br>Hoe oud was oom of tante 2 toen dit werd ontdekt?	Tussen 50 en 70 jaar<br>Ouder dan 70 jaar<br>Onbekend
11.1.2.4	***If 11.1.2 is bigger than 'Ja, 1 broer/zus' answer this question:***<br>In welk orgaan zat deze kanker? U kunt meerdere antwoorden aanvinken	Maag<br>Dunne darm (o.a. twaalfvingerige darm)<br>Alvleesklier<br>Galwegen<br>Nierbekken<br>Nieren<br>Hogere urinewegen<br>Eierstokken<br>Hersenen<br>Talgklier (goedaardig of kwaadaardig)<br>Baarmoeder<br>Baarmoederhals (Hierop wordt getest met het uitstrijkje bij het bevolkingsonderzoek)<br>Prostaat<br>Borst<br>Huid<br>Longen<br>Lymfeklier/leukemie<br>Slokdarm<br>Anders<br>Onbekend
11.1.2.5	***If 11.1.2 is bigger than 'Ja, 2 broers/zussen' answer this question:***<br>Hoe oud was oom of tante 3 toen dit werd ontdekt?	Jonger dan 50 jaar<br>Tussen 50 en 70 jaar<br>Ouder dan 70 jaar<br>Onbekend
11.1.2.6	***If 11.1.2 is bigger than 'Ja, 2 broers/zussen' answer this question:***<br>In welk orgaan zat deze kanker? U kunt meerdere antwoorden aanvinken	Maag<br>Dunne darm (o.a. twaalfvingerige darm)<br>Alvleesklier<br>Galwegen<br>Nierbekken<br>Nieren<br>Hogere urinewegen<br>Eierstokken<br>Hersenen<br>Talgklier (goedaardig of kwaadaardig)<br>Baarmoeder<br>Baarmoederhals (Hierop wordt getest met het uitstrijkje bij het bevolkingsonderzoek)

- [ ] Prostaat
- [ ] Borst
- [ ] Huid
- [ ] Longen
- [ ] Lymfeklier/leukemie
- [ ] Slokdarm
- [ ] Anders
- [ ] Onbekend

11.1.2.7	If 11.1.2 is bigger than 'Ja, 3 broers/zussen' answer this question:<br>Hoe oud was oom of tante 4 toen dit werd ontdekt?	○ Jonger dan 50 jaar<br>○ Tussen 50 en 70 jaar<br>○ Ouder dan 70 jaar<br>○ Onbekend
11.1.2.8	If 11.1.2 is bigger than 'Ja, 3 broers/zussen' answer this question:<br>In welk orgaan zat deze kanker? U kunt meerdere antwoorden aanvinken	☐ Maag<br>☐ Dunne darm (o.a. twaalfvingerige darm)<br>☐ Alvleesklier<br>☐ Galwegen<br>☐ Nierbekken<br>☐ Nieren<br>☐ Hogere urinewegen<br>☐ Eierstokken<br>☐ Hersenen<br>☐ Talgklier (goedaardig of kwaadaardig)<br>☐ Baarmoeder<br>☐ Baarmoederhals (Hierop wordt getest met het uitstrijkje bij het bevolkingsonderzoek)<br>☐ Prostaat<br>☐ Borst<br>☐ Huid<br>☐ Longen<br>☐ Lymfeklier/leukemie<br>☐ Slokdarm<br>☐ Anders<br>☐ Onbekend
