# Supplementary material for: Addition of an online, validated family history questionnaire to the Dutch FIT-based screening programme did not improve its diagnostic yield
Source: Br J Cancer. 2020 Apr 20;122(12):1865–71. doi: 10.1038/s41416-020-0832-8 (PMC7283285; doi:10.1038/s41416-020-0832-8)
Supplement: Supplementary file 2 — Supplementary Information Table 1 Referral criteria for genetic testing and colonoscopy surveillance [file 41416_2020_832_MOESM2_ESM.docx]

**Supplementary Information Table 1 Referral criteria for genetic testing and colonoscopy surveillance**

| **Referral criteria for suspicion of Lynch syndrome** |
| --- |
| 1. A patient with colorectal cancer or endometrial cancer <50 years |
| 2. A person with a first-degree relative with colorectal cancer or endometrial cancer <50 years |
| 3. A person with a family member with a mutation in one of the mismatch repair genes |
| 4. A person with at least three first- or second-degree relatives with colorectal cancer or a Lynch syndrome associated tumor^1^ all <70 years^2^ |
| 5. A patient with colorectal cancer with a synchronous or metachronous colorectal cancer <70 years |
| 6. A patient with colorectal cancer with a synchronous or metachronous Lynch syndrome associated tumor^1^  <70 years |
| 7. A patient with endometrial cancer with a synchronous or metachronous Lynch syndrome associated  tumor^1^ <70 years |
| 7. A patient with colorectal cancer with a first degree family member with colorectal cancer or a Lynch syndrome associated tumor^1^ <50 years |
| 8. A patient with endometrial cancer <70 years with a first degree family member with colorectal cancer or a Lynch syndrome associated tumor^1^ <50 years |
| 8. A patient with colorectal cancer or a Lynch syndrome associated tumor^1^ with at least two first- or second-degree relatives with colorectal cancer or a Lynch syndrome associated tumor^1^, all <70 years |

| **Referral criteria for suspicion of familial CRC syndrome** |
| --- |
| 9. A person with two first-degree family members with colorectal cancer 50-70 years^2^ |
| 10. A person with a first-degree relative with colorectal cancer 50-70 years and a second-degree relative with colorectal cancer <70 years^2^ |
| 11. A patient with colorectal cancer with a first-degree relative with colorectal cancer, both 50-70 years |
| 12. A patient with colorectal cancer 50-70 years and a second-degree relative with colorectal cancer <70 years |

| **Additional referral criteria** |
| --- |
| 13. A person with at least 2 first-degree relatives with colorectal cancer >70 years^2^ |
| 14. A person with at least 2 first-degree relatives with colorectal cancer, of whom one 50-70 years and the other >70 years^2^ |
| 15. Genetic testing performed, either Lynch/ FAP/ MAP confirmed or unknown |

^1^ Lynch syndrome associated tumor: endometrial cancer, stomach cancer, small bowel cancer, pancreatic cancer, biliary cancer, renal pelvis cancer, ureteral cancer, ovarian cancer, brain cancer, sebaceous gland adenoma or carcinoma.
^2^ Family members must be genetically related
